# Note from the editors: WHO declares mpox outbreak a public health emergency of international concern

**DOI:** 10.2807/1560-7917.ES.2024.29.33.240815v

**Published:** 2024-08-15

**Authors:** Eurosurveillance editorial team

**Affiliations:** 1 European Centre for Disease Prevention and Control (ECDC), Stockholm, Sweden

Yesterday, on 14 August 2024, the World Health Organization (WHO) declared the mpox outbreak in the Democratic Republic of the Congo (DRC) and a growing number of countries in Africa a public health emergency of international concern (PHEIC) under the International Health Regulations (IHR) (2005). The decision by Director-General Dr Tedros Adhanom Ghebreyesus was taken following the advice of an IHR Emergency Committee of independent experts, after reviewing data presented by experts from the WHO and affected countries earlier in the day [1]. The new strain of the monkeypox virus (MPXV), Clade Ib, that emerged last year in the DRC and has now been detected in neighbouring countries is one of the main reasons for the PHEIC decision by the WHO.

Dr Tedros said: *“The emergence of a new clade of mpox, its rapid spread in eastern DRC, and the reporting of cases in several neighbouring countries are very worrying. On top of outbreaks of other mpox clades in DRC and other countries in Africa, it’s clear that a coordinated international response is needed to stop these outbreaks and save lives.”*

This is the second PHEIC determination in 2 years related to mpox, following a declaration of a PHEIC in July 2022 for the global outbreak which was caused by MPXV Clade II and mainly affected men who have sex with men (MSM) in Europe and the Americas, and spread to more than 115 countries that had not reported cases previously [2]. The PHEIC was ended in May 2023, after a decline in cases worldwide.

*Eurosurveillance* has recently published articles related to the current outbreak. A rapid communication published on 14 March 2024 provided results from genomic sequencing of viral genomes to gain insight on the strains causing the current outbreak [3]. Authors showed, based on a phylogenetic analysis of near-to-complete MPXV genome sequences derived from six cases from the South Kivu province, that the MPXV belonged to a novel Clade I sub-lineage. They also showed that the outbreak strain genome lacks the target sequence of the probe and primers of a commonly used Clade I-specific real-time PCR. In another article published on 8 August 2024, authors described the laboratory validation and implementation of a new PCR assay to specifically detect Clade Ib viruses [4].

The European Centre for Disease Prevention and Control (ECDC) is providing regular updates related to mpox and to the current outbreak through a dedicated webpage [5]. ECDC also provides resources for healthcare professionals and basic information for the general public.
